# No Reason to Use Mesh in Groin Hernia Repair in Adolescents

**DOI:** 10.3389/jaws.2023.12336

**Published:** 2024-01-11

**Authors:** Hugin Reistrup, Siv Fonnes, Jacob Rosenberg

**Affiliations:** Center for Perioperative Optimization, Department of Surgery, Herlev Hospital, University of Copenhagen, Copenhagen, Denmark

**Keywords:** adolescents, teenagers, pediatric, groin hernia, inguinal hernia, hernia repair, mesh, non-mesh

## Abstract

Groin hernias are common and hernia repair is one of the most frequent surgical procedures performed worldwide. Despite this, there is no international guideline on the management of groin hernias in adolescents. Mesh reinforcement is used for repair in adults but not in young children. Adolescents, positioned between these age groups, pose unique challenges for surgeons due to their varying growth patterns. Placing a synthetic mesh in growing patients is a concern, particularly in relation to chronic pain. Traditionally, the hernia literature has defined adults as individuals aged 18 years and above. Considering that growth can continue until age 19, this review proposes a revised definition of adolescence for patients with groin hernias encompassing ages 10 to 19. Symptomatic groin hernias in adolescents should be repaired with an open non-mesh technique because of acceptable recurrence rates and the desire to avoid introducing synthetic foreign materials into young patients with ongoing growth potential. Watchful waiting is suggested for asymptomatic groin hernias, postponing repair until the adolescent has become a fully grown adult and symptoms from the hernia develop. Most groin hernias in adolescents are lateral hernias, but before pursuing a watchful waiting strategy in females, an ultrasound or magnetic resonance imaging scan is suggested to rule out the presence of a femoral hernia that may need repair.

## Introduction

The management of groin hernias in adolescents poses challenges for surgeons, particularly regarding the choice of surgical technique and timing of repair. There is no international guideline to support decision-making, and data in the literature are sparse with few studies dedicated to adolescents [1–13]. In young children and adults, the strategies for hernia management differ. Repair in young children is performed with open or laparoscopic non-mesh techniques. This approach has shown low recurrence rates of approximately 1% in children [14, 15]. In adults, repair is performed with a mesh approach with varying open and laparoscopic techniques as mesh repair has markedly lowered recurrence rates compared with non-mesh repair [16, 17]. While a conservative, non-operative watchful waiting strategy is feasible in male adults with asymptomatic inguinal hernias [18], it is not practiced in young children due to the risk of incarceration [14]. Adult females always require repair, preferably by laparoscopy [19, 20].

Adolescents differ from young children and adults. Growth patterns vary considerably between adolescents and, therefore, age is a poor measure of growth [21]. When repairing groin hernias in adolescents, surgeons must decide between mesh or non-mesh repair. In case of non-mesh repair, the risk of recurrence must be acceptable, and in case of mesh repair mesh-related complications must be acceptable. It seems counterintuitive to place a synthetic foreign body like a mesh, static in size, in the groin of adolescents who still have growth potential. Also, the prospect of living many years with a foreign body in the groin naturally raises concerns, and therefore, ideally, using mesh in this age group should perhaps be avoided.

The aim of this narrative review was to give an overview and recommendations based on current evidence on the management of groin hernias in adolescents aged 10–19 years.

## The Age of Adolescence

**FIGURE 1:**
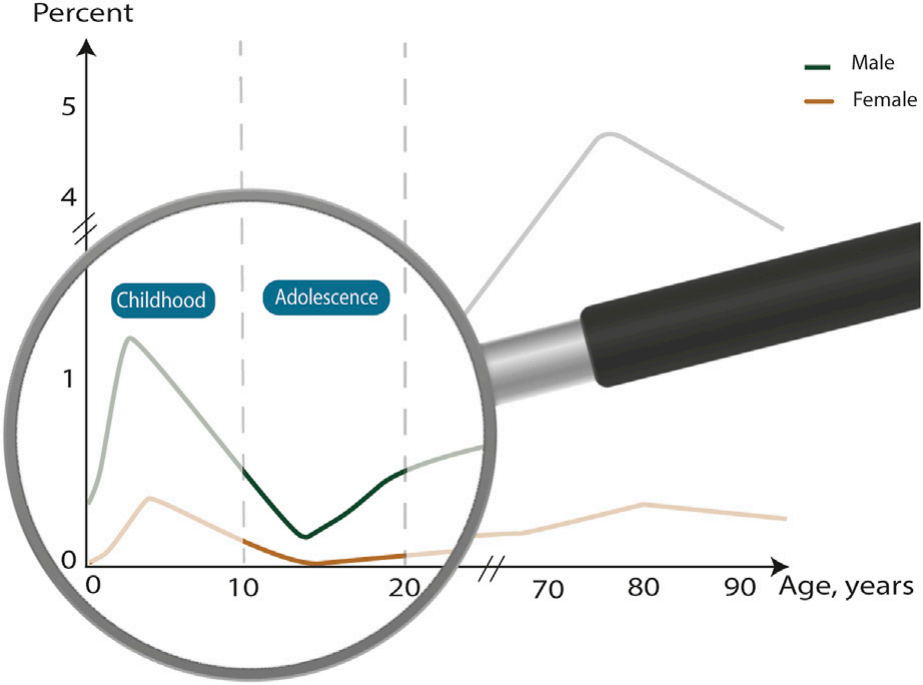
Classification of age groups in groin hernia patients and prevalence of repairs. Graph adapted from [31].

In the general hernia literature, adults are traditionally defined as 18 years and above [19], but there is no consensus on the definition of adolescents. In this review, we define adolescents as 10 to 19-year-olds (Figure 1). Definitions of age intervals vary in the general literature, but well-respected global institutions have clear definitions. The World Health Organization defines adolescence as 10–19 years [22], United Nations defines youth as 15–24 years [23], and a Lancet commission report on adolescent health and wellbeing followed a definition of age divided into 5-year age categories, where early adolescence was defined as 10–14 years, late adolescence as 15–19 years, and young adulthood as 20–24 years [24].

Adolescence is the phase between childhood and adulthood. Growth spurt is a central aspect of adolescence and is defined as a rapid increase in velocity of height and weight. Peak height velocity is a term used for the maximum rate of growth in stature taking place during the growth spurt, occurring about 2 years earlier in females than males, at 12 and 14 years, respectively [21, 25]. Final adult height can be expected to be reached at 19 years in males and at 17 years in females [26, 27]. In contrast to stature, there is no acceleration of growth of the pelvis during adolescence as both horizontal and vertical growth of the pelvis and growth of the inguinal ligaments are relatively linear from 0 to 19 years of age [28].

Placing a synthetic mesh in a growing groin worries surgeons, and when deciding between a mesh or non-mesh approach, hernia surgeons often use stature and growth spurt as measures of growth potential [11]. It is, however, important to be aware that these two do not necessarily reflect growth of the pelvis. Due to a potential growth of the pelvis until age 19, a definition of adolescence in the groin hernia literature from 10 to 19 years is therefore suggested in this review. For easy and user-friendly application in the everyday clinic, we suggest the same upper age limit of 19 years for both sexes.

## Groin Hernias in Adolescents

The etiology of groin hernias in adolescents is uncertain. One reason is the lack of data on hernia subtypes in this age group. In young children, 99% of groin hernias are lateral inguinal hernias [29], which are congenital and most often caused by a patent processus vaginalis [30]. Medial and femoral hernias are rare. In adults, 97% of groin hernias are inguinal hernias [31], and 60% of these are lateral [32]. Groin hernias in adults are primarily acquired, and occupational mechanical exposure is a known risk factor for lateral inguinal hernias in adults [33]. Few may be congenital hernias that have been asymptomatic for many years and, therefore, have not been repaired. The distribution of repairs is age-dependent, and the number of repairs is lowest in adolescents compared with all other age groups [31, 34]. The age distribution of hernia repairs is bimodal peaking before the age of 10 years and steadily increasing from 15 years until peaking again at 75 years (Figure 1). The lowest prevalence is found at 15 years. The prevalence of femoral hernias is different, increasing steadily throughout life and peaking at 80 years. Notably, a rapid increase starts around 30 years [31], which could be due to the widening of the pelvis related to women giving birth. Adolescents comprise a different group. Most of the congenital hernias have already been repaired in childhood, and groin hernias due to age-related factors affecting the groin have not yet had time to develop. A reason for the low occurrence of repairs in adolescents may be because surgeons choose to delay surgery until adolescents are fully grown, masking a higher occurrence of inguinal hernias than reported in the literature [11].

## Timing of Repair in Adolescents

Deciding when to repair a groin hernia in 10 to 19-year-olds can be challenging for surgeons. In most cases, a symptomatic groin hernia is an indication for repair regardless of the age of the patient. Watchful waiting is a term used in the hernia literature referring to a situation where a hernia is not repaired until it causes symptoms that require intervention. Delayed repair, conversely, entails planning a hernia repair at a later point in time, typically irrespective of symptoms. As groin hernias in adolescents will not disappear without intervention, an initial conservative strategy will practically always involve repair at some point. In adult males, about 70% of adult patients with asymptomatic or minimally symptomatic inguinal hernias following a conservative management strategy are repaired within 10 years due to the development of hernia-related symptoms [18]. Watchful waiting or delayed repair is not recommended in young children due to a 7% risk of incarceration and an even higher rate of 11% in preterm infants [14]. Unfortunately, data are not available on the time from diagnosis to repair. Watchful waiting is safe in adults with a low risk of 2%–3% of acute hernia-related operations [18]. No studies have assessed a conservative management strategy in adolescents. In a questionnaire study of 48 surgeons, most surgeons (70%) would repair an asymptomatic inguinal hernia in adolescents aged 13–18 years within 3 months, whereas some (20%) would wait and delay surgery until the adolescent was fully grown. Few (10%) would choose a watchful waiting strategy [35]. In a qualitative interview study of surgeons, statural growth was often the determining factor for timing of repair in adolescents [11]. Surgeons would postpone repair until patients were fully grown, at which point they would then perform a repair with a laparoscopic mesh-based technique. Using mesh in not fully grown adolescents worried most surgeons in the study.

## Mesh Versus Non-Mesh Repair in Adolescents

Anatomically, adolescents resemble adults more than young children, but still, adolescence is a period of growth, unlike adulthood. As growth stages vary between adolescents and age is a poor measure of growth, the decision on when and whether to treat adolescents with groin hernias as either young children or adults can be challenging. Mesh repair is the standard approach in adults [19] as it lowers recurrence rates [16, 17], and a laparoscopic approach seems to be increasingly used [36]. In children, a mesh is not used as simple high ligation results in low recurrence rates of about 1% [14, 15]. While there will be national differences, and the use of minimally invasive surgery is increasing in many countries, open repair for inguinal hernias in children still seems to be preferred over laparoscopy [37, 38].

Data comparing mesh and non-mesh repairs in adolescents are sparse, as are long-term follow-up data. A meta-analysis of 4,000 groin hernia repairs showed that mesh was seldom used in adolescents, and repairs were most often performed with open techniques [10]. Most of the studies were small retrospective cohort studies while only a few were prospective, and there were no high-quality randomized controlled trials on this subject. The meta-analysis showed low incidences of recurrence (<2%) across both mesh and non-mesh techniques. Also, the rate of chronic pain seemed acceptable ranging from 0% to 11% across all surgical techniques. A case-based survey showed that surgeons had varying surgical approaches in adolescents depending on whether they were mostly handling paediatric or adult patients [12]. Paediatric surgeons used high ligation while adult surgeons used either mesh or non-mesh techniques other than high ligation, suggesting inconsistency in treatment depending on which surgeon is treating the adolescent.

## Discussion

**FIGURE 2:**
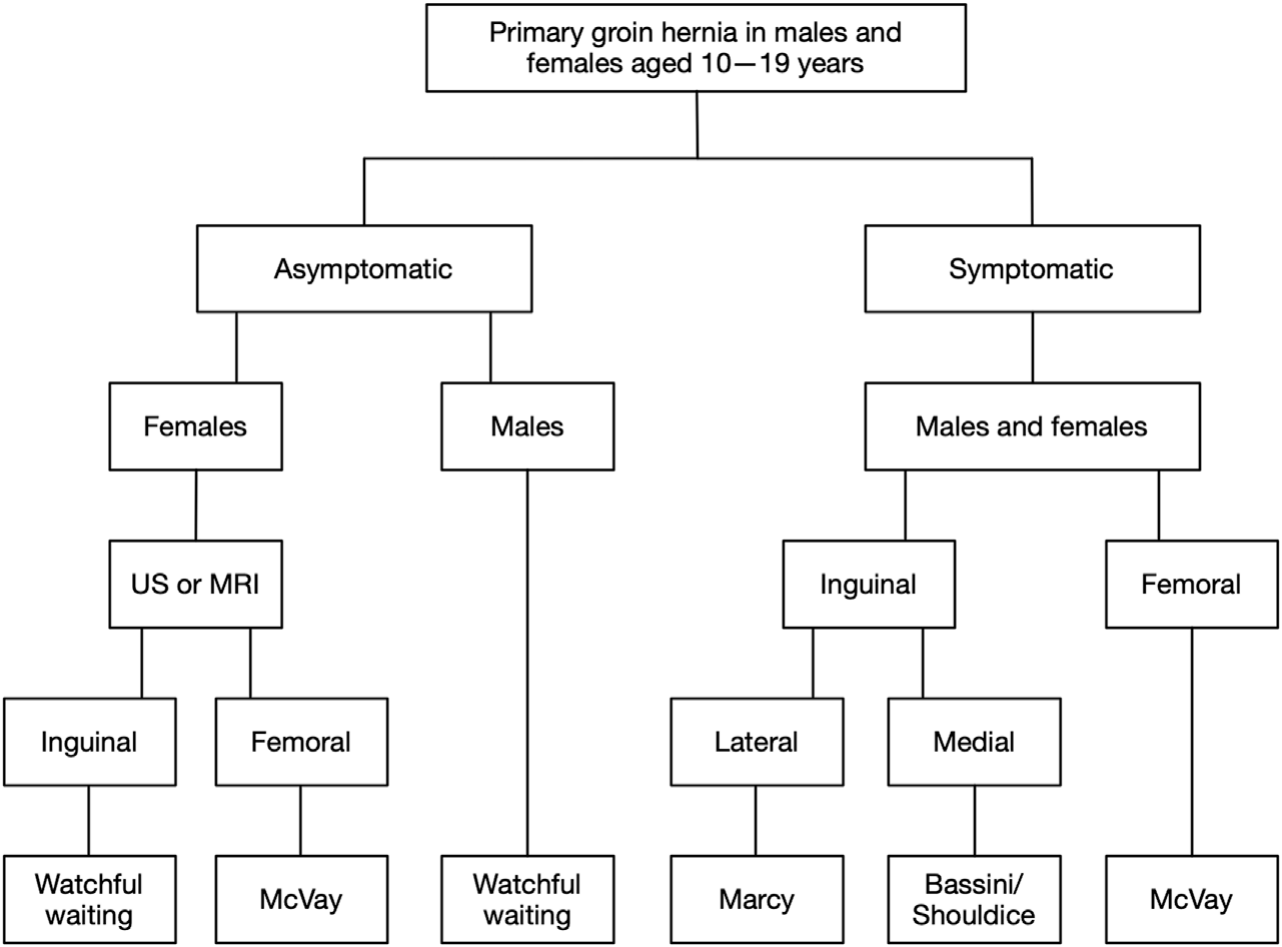
Flowchart of suggested management strategy for groin hernias in adolescents. For symptomatic hernias, open non-mesh repair is recommended for males and females due to low recurrence rates, with surgical technique depending on hernia subtype. For asymptomatic hernias, watchful waiting is recommended for males, postponing repair until the patient is a fully grown adult and symptoms from the hernia develop. For females with asymptomatic hernias, an ultrasonography or magnetic resonance imaging scan may be performed to exclude the presence of a rare femoral hernia before pursuing a watchful waiting strategy. US, ultrasonography; MRI, magnetic resonance imaging.

Based on the available evidence, there seems to be no need to use mesh in groin hernia repair in adolescents aged 10–19 years. Recurrence rates are low after non-mesh repairs, and little is known about the long-term consequences of placing a synthetic mesh in the groin of these young patients. Consequently, symptomatic hernias in both male and female adolescents should be repaired with an open non-mesh technique. A conservative management strategy has not been investigated, but data suggest that surgeons are already using this approach for asymptomatic hernias to delay repair until adolescents are fully grown. Therefore, a conservative, non-operative watchful waiting strategy is recommended for asymptomatic groin hernias in males, while preoperative imaging is recommended for females to exclude the presence of a rare femoral hernia (Figure 2).

There is a need for consensus on the definition of adolescence in the groin hernia literature. We suggest a definition of adolescence from 10 to 19 years for both males and females (Figure 1). Traditionally, the hernia literature defines adults as 18 years and above, but there does not seem to be a well-founded anatomical or physiological reasoning for this lower age limit. On the contrary, growth of the pelvis and groin in healthy and well-nourished individuals can be expected to continue up to age 19 in both sexes [28]. To avoid implanting a synthetic foreign body like a mesh in a groin that is not fully grown, it is probably better to refrain from using mesh in patients who still have growth potential. In ventral hernia repair in women with subsequent pregnancies, mesh repair resulted in an increased risk of chronic pain compared with non-mesh repair [39]. This might be due to the tension on the mesh as the abdominal wall is strained during pregnancy. In theory, the same principle might apply to a mesh implanted in the groin of females with subsequent pregnancies. This could be a further argument for avoiding meshes in female adolescents.

Mesh-related complications are well known following hernia repair in adults [40]. Data on long-term follow-up after mesh repair in adolescents are too sparse for any firm conclusions on this surgical approach [10]. Especially, there is a lack of data on postoperative complaints like chronic pain, sexual dysfunction, and effects on fertility, as well as overall health economic implications. Data on groin hernia repair in young adults might be an indicator of expected outcomes in adolescents. In the latest updated international guideline on groin hernia management [41], annulorrhaphy is suggested as an alternative to mesh repair in young men with small indirect inguinal hernias. A large-scale questionnaire study on 2,612 adult patients comparing open mesh (Lichtenstein) and non-mesh (Shouldice and Marcy) repair of indirect inguinal hernias showed that pain was more frequent in patients younger than 40 years of age, but the study did not find a difference in pain between the surgical approaches [42]. A prospective study on 669 adult patients with inguinal hernias comparing total extraperitoneal (TEP) and Lichtenstein repair (both mesh-based approaches) also found that pain was more frequent in patients younger than 40 years of age [43]. A nationwide register study investigating fertility concluded that bilateral open and laparoscopic mesh repairs did not impair fertility in 18–30 years-old males [44]. Looking at recurrence, a database study on adults found that young male patients aged 18–29 years had lower reoperation rates for recurrence after non-mesh repair of indirect inguinal hernias compared with all other age groups [45]. Biological, resorbable meshes are not routinely used in adult groin hernia repair, but the potential applicability in adolescents may be interesting due to the resorbable property of the mesh. However, the current lack of data prohibits the recommendation of biological meshes for adolescents [10]. Also, current data do not permit recommendations for a treatment strategy based on the size of the hernia defect.

In Figure 2, a suggested management algorithm is presented. For asymptomatic groin hernias, watchful waiting is recommended for adolescent males. The asymptomatic hernias can then be repaired when the adolescents have become fully grown adults and symptoms develop from the hernia. There is no data in the literature on the timing of repair in adolescents, but surgeons often choose a conservative strategy suggesting it is safe from an experience-based perspective [11, 35]. Females are a special entity as femoral hernias are more common in females than males [31], and femoral hernias have a higher risk of emergency repair compared with inguinal hernias in adults (36% vs. 5%) [46]. Still, femoral hernia repairs are rare in young children and adolescents, with a significant increase in prevalence starting around age 30 [31]. In the management of groin hernias in adults, the mantra is that all groin hernias in females should be considered potential femoral hernias. Therefore, a laparoscopic mesh repair is recommended as mesh lowers the risk of recurrence [19, 20]. In adolescent females with groin hernias, the risk of the hernia being a femoral hernia is very low. In both females and males, only 2% of the total number of femoral hernia repairs were performed before the age of 20 [31]. As a precautionary measure, before pursuing a watchful waiting strategy, a diagnostic ultrasonography performed by an experienced examiner, or, alternatively, a magnetic resonance imaging scan, could be performed to exclude the presence of a rare femoral hernia.

For symptomatic groin hernias, open non-mesh repair is recommended for both males and females. The choice of open surgical technique depends on the hernia subtype (Figure 2). Recurrence rates after non-mesh repair in adolescents seem acceptably low (<2%), though data are relatively sparse [10]. Also, postoperative mesh-related complications can be worrying, and a potential mesh removal is difficult and carries a risk of damage to nerves and vessels. In case of recurrence after a primary open non-mesh repair, a re-repair is easily performed by laparoscopy with mesh implantation. As stated earlier, femoral hernias are an issue. Still, a groin hernia in a female is most likely an indirect inguinal hernia, especially at a young age.

With the increasing use of laparoscopic repairs, experience and skills in open techniques will naturally diminish for future surgeons [36]. Groin hernias in adolescents will in most cases be lateral inguinal hernias in both sexes, and for these, a simple annulorrhaphy (Marcy repair) is sufficient. In case of medial or femoral hernias, other techniques apply (Figure 2). Surgeons repairing groin hernias in adolescents must therefore have sufficient experience in managing the variety of open techniques applicable, but it is not realistic to expect that all future surgeons have this level of experience in open repairs. Due to this, it is important to ensure that the proper surgical expertise is present when performing open repairs in adolescents. If this is not possible, patients should be referred to dedicated hernia centres.

There are limitations to this review, mainly related to its mini review format. A formal and comprehensive literature search was not conducted, and the study was not reported in accordance with a reporting guideline since, to our knowledge, none exists for mini reviews. Furthermore, the scarcity of data in the literature on this subject impedes firm, evidence-based conclusions. Still, some evidence does exist, and adolescents with groin hernias do seek consultation with surgeons in the clinic. Therefore, there is a need for guidance based on the best available evidence.

Conducting randomized controlled trials to compare mesh and non-mesh repair for groin hernias in adolescents may not be feasible due to the low prevalence of the condition in this population. Instead, large register-based cohort studies with sufficiently long follow-up may be a more suitable method. If such studies demonstrate acceptable rates of recurrence after non-mesh repair in adolescents aged 10–19 years, further investigations on mesh repair in this population may be unnecessary. Also, conducting questionnaire studies on postoperative complaints such as chronic pain and sexual dysfunction would provide further valuable information on potential harms. Investigations on the safety and feasibility of watchful waiting are also needed. When including adolescents aged 10–19 years in studies on groin hernias, we encourage researchers to report subgroup analyses on this population if feasible.

## Conclusion

Data on the management of groin hernias in adolescents are sparse, but the risk of recurrence seems low after open non-mesh repair. Therefore, we recommend avoiding meshes in adolescents aged 10–19 years and repair symptomatic groin hernias in males and females with an open non-mesh approach. For asymptomatic hernias, we recommend watchful waiting in adolescent males awaiting repair until the patient is a fully grown adult and symptoms develop from the hernia. In adolescent females with asymptomatic groin hernias, preoperative imaging may be performed to exclude the presence of a rare femoral hernia that could argue for operation rather than watchful waiting.
